# Hsa-miR-196a2 Rs11614913 Polymorphism Contributes to Cancer Susceptibility: Evidence from 15 Case-Control Studies

**DOI:** 10.1371/journal.pone.0018108

**Published:** 2011-03-31

**Authors:** Haiyan Chu, Meilin Wang, Danni Shi, Lan Ma, Zhizhong Zhang, Na Tong, Xinying Huo, Wei Wang, Dewei Luo, Yan Gao, Zhengdong Zhang

**Affiliations:** 1 Jiangsu Key Lab of Cancer Biomarkers, Prevention & Treatment, Cancer Center, Nanjing Medical University, Nanjing, China; 2 The Key Laboratory of Modern Toxicology, Ministry of Education, Nanjing Medical University, Nanjing, China; 3 Department of Molecular & Genetic Toxicology, School of Public Health, Nanjing Medical University, Nanjing, China; Baylor College of Medicine, United States of America

## Abstract

**Background:**

MicroRNAs (miRNAs) are a family of endogenous, small and noncoding RNAs that negatively regulate gene expression by suppressing translation or degrading mRNAs. Recently, many studies investigated the association between hsa-miR-196a2 rs11614913 polymorphism and cancer risk, which showed inconclusive results.

**Methodology/Principal Findings:**

We conducted a meta-analysis of 15 studies that included 9,341 cancer cases and 10,569 case-free controls. We assessed the strength of the association, using odds ratios (ORs) with 95% confidence intervals (CIs). Overall, individuals with the TC/CC genotypes were associated with higher cancer risk than those with the TT genotype (OR = 1.18, 95% CI = 1.03–1.34, *P*<0.001 for heterogeneity test). In the stratified analyses, we observed that the CC genotype might modulate breast cancer risk (OR = 1.11, 95%CI = 1.01–1.23, *P*_heterogeneity_ = 0.210) and lung cancer risk (OR = 1.25, 95%CI = 1.06–1.46, *P*_heterogeneity_ = 0.958), comparing with the TC/TT genotype. Moreover, a significantly increased risk was found among Asian populations in a dominant model (TC/CC versus TT, OR = 1.24, 95% CI = 1.07–1.43, *P*_heterogeneity_ = 0.006).

**Conclusions:**

These findings supported that hsa-miR-196a2 rs11614913 polymorphism may contribute to the susceptibility of cancers.

## Introduction

MicroRNAs (miRNAs) are a class of noncoding evolutionarily conserved small RNA molecules with ∼22 nucleotides in length that inhibit gene expression. Mature miRNAs operate via sequence-specific interactions with the 3’ untranslated region (UTR) of cognate mRNA targets, causing suppression of translation and mRNA decay [1], [2]. MiRNAs play important roles in the etiology of many human diseases through post-transcriptionally regulating the expression of approximately one third of all human genes [3].

At present, miRNAs have been involved in the etiology, progression and prognosis of cancer [4]. In 2008, Hu *et al.* reported hsa-miR-196a2 might be a prognostic biomarker for non-small cell lung cancer [5]. Moreover, Pineau *et al.* found that the expression levels of some miRNAs change gradually during the progression of liver disease and miR-221 is capable of stimulating tumor growth possibility [6].

Single nucleotide polymorphisms (SNPs) occurring in miRNAs are novel sources of genetic variation that may contribute to cancer risk [7]. Recently, many studies research the association between rs11614913 SNP in hsa-miR-196a2 and cancer susceptibility, such as breast cancer [8]–[10], lung cancer [11], [12], hepatocellular carcinoma (HCC) [13], [14], gastric cancer [15], [16], Esophageal Cancer (EC) [17], [18] and others [19–2]. In addition, Gao *et al.* performed a meta-analysis of hsa-miR-196a2, suggesting that hsa-miR-196a2 rs11614913 polymorphism was associated with an increased breast cancer risk [23].

However, the results of these studies remain conflicting rather than conclusive. Considering the important role of hsa-miR-196a2 rs11614913 in the carcinogenic process, we performed a meta-analysis on all eligible case-control studies to estimate the overall cancer risk and to quantify the potential between-study heterogeneity.

## Materials and Methods

### Literature search and data extraction

In order to identify the relative papers on hsa-miR-196a2 rs11614913 T>C polymorphism and cancer risk, we performed a systematic search from PubMed and other studies in Public Domain updated to December 20, 2010. The search was limited to English language papers, using the key words ‘miR-196a2’ or ‘rs11614913’, ‘polymorphism’ and ‘cancer’. In addition, studies were identified by a manual search of the reference lists of reviews and retrieved studies. If there were detailed allele frequency data for the hsa-miR-196a2 polymorphism with cancer risk in a case-control design, studies were selected. The data for this analysis were available from 15 case-control studies, including 9,341 cases with different types of cancers and 10,569 controls.

Two investigators independently extracted the data and reached consensus on all items. The following information was sought from each publication: the first author’s name, year of publication, country of origin, ethnicity, cancer type, source of control (population- or hospital-based controls), genotyping method and number of cases and controls. For study including subjects of different countries of origin group, we extracted data separately [10]. Different ethnic descents were categorized as European, Asian or mixed (composed of an admixture of different ethnic groups). One study without detailed genotyping data was excluded in the analyses [24].

### Statistical analysis

The departure of frequencies of hsa-miR-196a2 polymorphism from expectation under Hardy-Weinberg equilibrium (HWE) was assessed by χ^2^ test in controls. The strength of the association between the hsa-miR-196a2 polymorphism and cancer risk was measured by odds ratios (ORs) with 95% confidence intervals (CIs). The statistical significance of the summary OR was determined with the Z-test. Pooled estimates of the OR were obtained by calculating a weighted average of OR from each study. We first estimated the risks of the variant genotype TC and CC, compared with the wild-type TT homozygote, and then evaluated the risks of TC/CC versus TT and CC versus TC/TT, assuming dominant and recessive effects of the variant C allele, respectively. Stratified analyses were also performed by cancer types (if one cancer type contained one individual study, it was combined into other cancers group), ethnicity and source of controls.

Statistical heterogeneity between studies was assessed with χ^2^ based Q test, and the heterogeneity was considered significant when *P*<0.10 [25]. The fixed-effects model and the random-effects model, based on the Mantel-Haenszel method and the DerSimonian and Laird method, respectively, were used to pool the data from different studies [26], [27]. In the absence of heterogeneity, these models provide similar results; otherwise, the random-effects model is more appropriate. Sources of heterogeneity were determined by using random-effects meta-regression models with restricted maximum likelihood estimation. The interstudy variance (τ^2^) was used to quantify the degree of heterogeneity between studies, and the percentage of τ^2^ was used to describe the extent of explained heterogeneity of the characteristics [28].

Publication bias was evaluated with the funnel plot and the linear regression asymmetry test by Egger *et al* [29]. *P*<0.10 was used as an indication for the presence of potential publication bias. All analyses were done with Stata software (version 8.2; StataCorp LP, College Station, TX, USA), and all tests were two-sided.

## Results

### Characteristics of studies

**Figure 1:**
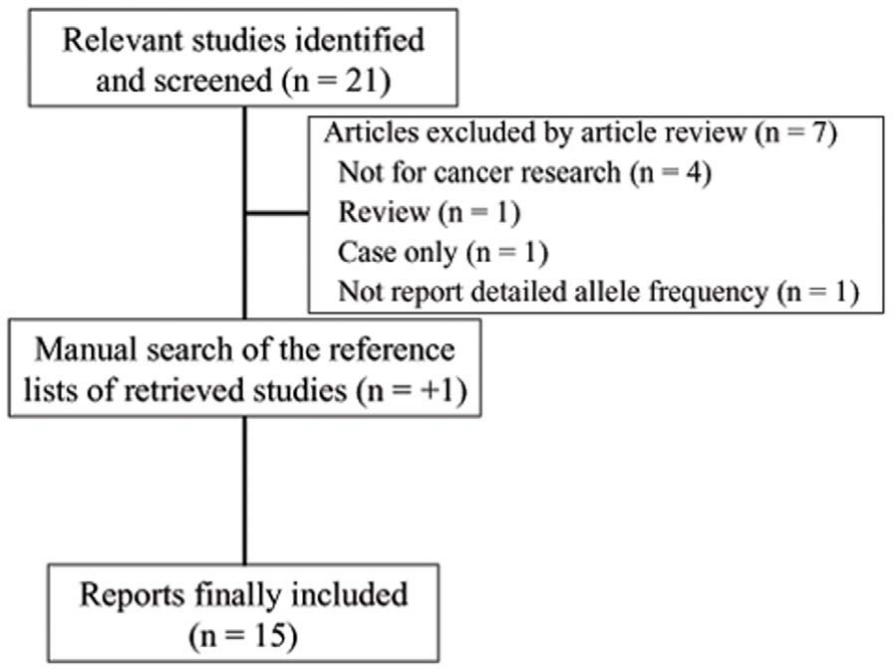
Studies identified with criteria for inclusion and exclusion.

There were 22 articles relevant to the search words and manual search (Figure 1). Among these, 15 studies that included a total of 9,341 cancer cases and 10,569 controls appeared to meet the inclusion criteria and were subjected to further examination. We excluded seven studies (four were not for cancer research, one was review, one was case only study and one did not report detailed allele frequency data). The selected study characteristics are summarized in Table 1. All studies were case-control studies, including four breast cancer studies, two lung cancer studies, two gastric cancer studies, two HCC studies, two esophageal cancer and the others were categorized into the “ther cancers”group. Cancers were histologically or pathologically in most studies. There were nine studies of Asian descent, six studies of European descent and one study of mixed ethnicity descent. A classic polymerase chain reaction–restriction fragment length polymorphism (PCR-RFLP) assay was conducted in eight of the 15 studies (Table 1). The distribution of genotypes in the controls was all in agreement with HWE for all except one study (*P* = 0.002) [21].

**Table 1:** Characteristics of literatures included in the meta-analysis.

First author	Year	Country	Cancer type	Ethnicity	Genotyping	Source of control	Sample size (case/control)
Hu	2008	China	Breast cancer	Asian	PCR-RFLP	PB	1009/1093
Ye	2008	USA	EC	European	SNPlex assay	HB	307/338
Tian	2009	China	Lung cancer	Asian	PCR-RFLP	PB	1058/1035
Hoffman	2009	USA	Breast cancer	Mixed	MassArray multiplex (sequenom)	HB	426/466
Peng	2010	China	Gastric cancer	Asian	PCR-RFLP	HB	213/213
Qi	2010	China	HCC	Asian	PCR–LDR	HB	361/391
Dou	2010	China	Glioma	Asian	PCR–LDR	HB	643/656
Li	2010	China	HCC	Asian	PCR-RFLP	HB	310/222
Wang	2010	China	EC	Asian	SNaPshot assay	HB	458/489
Catucci	2010	Germany	Breast cancer	European	Taqman	HB	1101/1496
		Italy	Breast cancer	European	Taqman	HB	751/1243
Srivastava	2010	India	Gallbladder cancer	European	PCR-RFLP	PB	230/230
George	2010	India	Prostate cancer cancer	European	PCR-RFLP	HB	159/230
Okubo	2010	Japan	Gastric cancer	Asian	PCR-RFLP	HB	552/697
Kim	2010	Korea	Lung cancer	Asian	Fuorescence	HB	654/640
Liu	2010	USA	SCCHN	European	PCR-RFLP	HB	1109/1130

EC: Esophageal Cancer; HCC: Hepatocellular Carcinoma; SCCHN: Squamous Cell Carcinoma of the Head and Neck; PCR-RFLP: Polymerase Chain Reaction-restriction Fragment Length Polymorphism; PCR–LDR: Polymerase Chain Reaction–Ligation Detection Reaction; Fuorescence: Labeled Hybridization Probes; PB, Population Based; HB, Hospital Based.

### Quantitative synthesis

**Figure 2:**
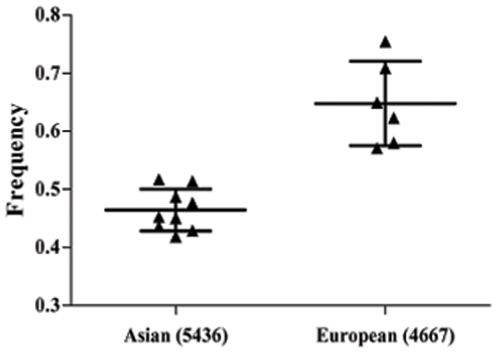
Frequencies of the variant alleles (C allele) among controls stratified by ethnicity. Black trangle ▴ represents each included study.

There was a variation in the C allele frequency of the hsa-miR-196a2 rs11614913 T>C polymorphism among the controls across different ethnicities, ranging from 0.42 to 0.75. For European controls, the C allele frequency was 0.65 (95% CI = 0.57–0.72), which was higher than that in Asian controls (0.46, 95%CI = 0.44–0.49, *P*<0.001; Figure 2).

Overall, we observed the hsa-miR-196a2 rs11614913 polymorphism could increase the risk of cancer in all genetic models except recessive model, when all the eligible studies were pooled into the meta-analysis. As shown in Table 2, the TC heterozygote (OR = 1.16, 95% CI = 1.02-1.32, *P*_heterogeneity_ = 0.001) and variant CC homozygote (OR = 1.22, 95% CI = 1.04–1.44, *P*_heterogeneity_ <0.001) were associated with significantly increased risks of all types of cancers compared with wild-type homozygote (TT). Significant main effect was also observed in dominant model (OR = 1.18, 95% CI = 1.03–1.34, *P*_heterogeneity_ <0.001). However, we did not observe the similar effect in recessive model (OR = 1.06, 95% CI = 0.95–1.18, *P*_heterogeneity_ <0.001; Table 2).

**Table 2:** Meta-analysis for the hsa-miR-196a2 rs11614913 polymorphism and cancer risk.

Variables	n^a^	TC versus TT		CC versus TT		TC/CC versus TT (dominant)		CC versus TC/TT (recessive)	
		OR (95% CI)	*P* b	OR (95% CI)	*P* b	OR (95% CI)	*P* b	OR (95% CI)	*P* b
Total	16	1.16 (1.02–1.32)	0.001	1.22 (1.04–1.44)	<0.001	1.18 (1.03–1.34)	<0.001	1.06 (0.95–1.18)	<0.001
Cancer types									
Breast cancer	4	1.15 (1.01–1.31)	0.162	1.30 (1.01–1.68)	0.028	1.22 (1.00–1.50)	0.062	1.11 (1.01–1.23)	0.210
Lung cancer	2	1.08 (0.92–1.27)	0.493	1.31 (1.09–1.58)	0.674	1.15 (0.99–1.34)	0.470	1.25 (1.06–1.46)	0.958
Gastric cancer	2	1.07 (0.85–1.34)	0.848	1.25 (0.94–1.65)	0.306	1.12 (0.90–1.39)	0.698	1.22 (0.97–1.55)	0.162
HCC	2	1.10 (0.85–1.43)	0.125	1.25 (0.65–2.39)	0.040	1.17 (0.72–1.89)	0.051	1.13 (0.87–1.46)	0.137
EC	2	1.19 (0.29–4.81)	<0.001	1.22 (0.26–5.72)	<0.001	1.20 (0.28–5.11)	<0.001	1.05 (0.63–1.74)	0.021
Other cancers	4	1.14 (1.06–1.22)	0.391	0.94 (0.78–1.12)	0.744	1.09 (0.93–1.27)	0.625	0.81 (0.71–0.93)	0.322
Ethnicities									
Asian	9	1.20 (1.03–1.39)	0.015	1.33 (1.09–1.62)	0.002	1.24 (1.07–1.43)	0.006	1.17 (1.02–1.34)	0.037
European	6	1.01 (0.81–1.28)	0.040	0.97 (0.85–1.12)	0.121	0.98 (0.79–1.22)	0.046	0.91 (0.79–1.03)	0.099
Mixed	1	1.80 (1.16–2.80)	-	2.15 (1.37–3.38)	-	1.95 (1.27–2.98)	-	1.34 (1.02–1.75)	-
Source of controls									
Population-based	3	1.11 (0.97–1.28)	0.503	1.30 (1.10–1.54)	0.743	1.16 (1.02–1.33)	0.729	1.09 (0.84–1.42)	0.038
Hospital-based	13	1.17 (1.00–1.38)	<0.001	1.22 (0.99–1.49)	<0.001	1.19 (1.01–1.40)	<0.001	1.05 (0.93–1.18)	0.002

a Number of comparisons.

b *P* value of Q-test for heterogeneity test. Random-effects model was used when *P* value for heterogeneity test <0.10; otherwise, fix-effects model was used.

HCC: hepatocellular carcinoma; EC: Esophageal Cancer.

We then evaluated the effects of hsa-miR-196a2 rs11614913 T>C according to specific cancer types, different ethnicities and different source of control. As show in Table 2, individuals carrying the CC genotype could elevate breast cancer risk (CC versus TT, OR = 1.30, 95%CI = 1.01–1.68, *P*_heterogeneity_ = 0.028; CC versus TC/TT, OR = 1.11, 95% CI = 1.01–1.23, *P*_heterogeneity_ = 0.210) and lung cancer risk (CC versus TT, OR = 1.31, 95% CI = 1.09–1.58, *P*_heterogeneity_ = 0.674; CC versus TC/TT, OR = 1.25, 95% CI = 1.06-1.46, *P*_heterogeneity_ = 0.958) compared with those with TT or TC/TT genotypes. In addition, we found that individuals with TC genotype had an increased risk of breast cancer, comparing with TT genotype (OR = 1.15, 95% CI = 1.01–1.31, *P* = 0.162 for heterogeneity test). In recessive genetic model, a significantly decreased risk was found for other cancers (OR = 0.81, 95% CI: 0.71–0.93, *P* = 0.748 for heterogeneity test; Table 2).

In the stratified analysis by ethnicity, significantly increased risks were found in Asians (TC versus TT, OR = 1.20, 95% CI = 1.03–1.39, *P*_heterogeneity_ = 0.015; CC versus TT, OR = 1.33, 95% CI = 1.09–1.62, *P*_heterogeneity_ = 0.002; TC/CC versus TT, OR = 1.24, 95% CI = 1.07–1.43, *P*_heterogeneity_ = 0.006; CC versus TC/TT, OR = 1.17, 95% CI = 1.02–1.34, *P*_heterogeneity_ = 0.037) in all genetic models tested. However, no significant association was observed in Europeans (Table 2). According to source of controls, significant effects were observed in both population-based studies (TC/CC versus TT, OR = 1.16, 95% CI = 1.02–1.33, *P*_heterogeneity_ = 0.729) and hospital-based studies (TC/CC versus TT, OR = 1.19, 95% CI = 1.01–1.40, *P*_heterogeneity_ <0.001; Table 2).

### Test of heterogeneity

Heterogeneity between studies was observed in overall comparisons and subgroup analyses. Then, we assessed the source of heterogeneity for recessive model (CC versus TC/TT) by cancer type, ethnicity and source of controls. As a result, cancer type (χ^2^ = 22.55, df = 5, *P*<0.001) and ethnicity (χ^2^ = 14.46, df = 2, *P* = 0.001) but not source of controls (χ^2^ = 2.08, df = 1, *P* = 0.150) were found to contribute to substantial heterogeneity. Furthermore, meta-regression analyses revealed that cancer type can explain 25.5% (CC versus TC/TT, *P* = 0.025) of the τ^2^.

### Publication bias

**Figure 3:**
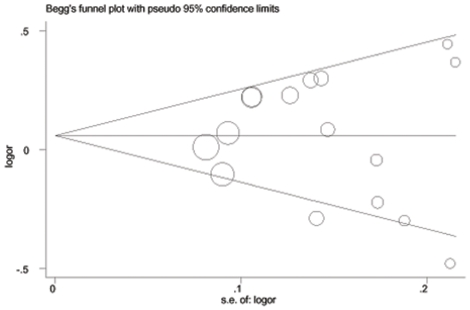
Begg's funnel plot for publication bias test (CC versus TC/TT). Each point represents a separate study for the indicated association. Log[or], natural logarithm of OR. Horizontal line, mean effect size.

Begger’s funnel plot and Egger’s test were performed to assess the publication bias of included studies. As shown in Figure 3, the shapes of the funnel plots did not reveal any evidence of obvious asymmetry in all comparison models. Then, Egger’s test was used to provide statistical evidence of funnel plot symmetry. The results still did not show any evidence of publication bias (*t* = −0.19, *P* = 0.853 for CC versus TC/TT).

## Discussion

In the present study, hsa-miR-196a2 rs11614913 T>C polymorphism was found to be associated with an significantly increased cancer risk in the variant TC heterozygote, CC homozygote, and TC/CC genotype as opposed to the TT wild-type homozygote, including 9,034 cancer cases and 10,231 controls.

The biogenesis of miRNAs is related to a complex protein system, such as dicer and drosha [30]. MiRNAs participate in crucial biological process, including development, differentiation, apoptosis and proliferation [31], [32]. Genetic variations in the miRNA genes could potentially influence the processing or target selection of miRNAs [33]. Recently, Ryan *et al.* conducted a review about genetic variation in miRNA for the cancer research, suggesting that the variations in miRNA might be related to the risk of cancer [34]. Tian *et al*. observations suggested that hsa-miR-196a2 rs11614913 polymorphism could contribute to the risk of lung cancer [11]. Hu *et al.* thought that rs11614913 polymorphism might be a prognostic biomarker for lung cancer [5]. Interestingly, in 2010, so far there had been eleven published studies about the association between hsa-miR-196a2 rs11614913 polymorphism and cancer risk. Hsa-miR-196a2 may be a hot concern to predict cancer susceptibility. Given the important role of that rs11614913 polymorphism locating in the hsa-miR-196a2 3′ mature sequence affects the maturation and effect of target mRNA possibility [34], it is biologically plausible that genetic variation of hsa-miR-196a2 may modulate cancer susceptibility.

Consistent with Gao *et al* observations, our results showed that the CC genotype may increase breast cancer risk in a C allele dose-response manner [23]. Hu *et al*. showed that expression levels of mature of hsa-mir-196a2 were increased in rs11614913 CC in the human lung cancer tissues and binding assays revealed that the rs11614913 could affect binding of mature hsa-miR-196a2 to its target mRNA [5]. Moreover, we also found that individuals carrying CC genotype might increase lung cancer susceptibility. However, the similar association was not observed in HCC, gastric cancer and EC patients. One factor that would contribute to the discrepancy between different studies is that this polymorphism might play a different role in different cancer sites. Landgraf *et al*. found that 3p hsa-miR-196a2 was detectable in the MCF-7 breast adenocarcinoma cell line [35]. Hu *et al*. reported that hsa-miR-196a2 rs11614913 T>C located in hsa-mir-196a2′ 3p mature miRNA regions, was associated with an increased risk of breast cancer [9]. In addition, rs11614913 T>C was shown to be associated with lung cancer risk, through altering the expression of mature miR-196a and binding activity of target mRNA [5]. We observed that there was a significantly decreased risk of other cancers in the recessive model. Although the exact mechanism for this inverse association was not clear, carcinogenic mechanisms may differ for different tumor sites and the hsa-miR-196a2 genetic variants may exert varying effects in different cancers. In addition, other cancers is composed of four different tumor studies. Therefore, the results should be interpreted with caution.

In population level, we found that individuals with variant C allele could increase cancer susceptibility in Asians but not in Europeans, suggesting a possible role of ethnic differences in genetic background and the environment they lived in [36]. When stratifying the source of control, significant associations were observed in hospital-based and population-based controls. This may result from most of the included studies matching age, sex and residential area and sample size >500 to control selection bias.

To identify the source of heterogeneity, we stratified the studies according to ethnicity, cancer type and source of control. We found the sources of heterogeneity were from ethnicity and cancer type, suggesting population and cancer specific playing important roles.

One of the strengths of our meta-analysis was that numbers of cases and controls were pooled from each included studies, which significantly increased the statistical power. Second, according our selection criteria, the quality of studies included in our meta-analysis was satisfactory. Third, furthermore, on the basis of our study, functional variants of hsa-miR-196a2 might be conducted and replicate these observations, so that it might find a novel mechanism to predict the risk of cancer. Some limitations exist in our meta-analysis. First, we pooled the data based on unadjusted information, while a more precise analysis needs to be conducted if individual data are available. Second, lacking the origin data of including studies limited the further evaluation of the potential interactions, because gene-environment interactions may modulate the cancer susceptibility. Third, studies published in English were included and selection bias could have occurred.

In conclusion, our meta-analysis suggested that the hsa-miR-196a2 rs11614913 polymorphism may contribute to genetic susceptibility for increased cancer risk. We observed the similar association in Asian populations but not in Europeans. Future larger population and functional study should be performed to validate these results and need to replicate in Africans.

## References

[1] Ambros V (2004). The functions of animal microRNAs.. Nature.

[2] Bartel DP (2009). MicroRNAs: target recognition and regulatory functions.. Cell.

[3] Lewis BP, Burge CB, Bartel DP (2005). Conserved seed pairing, often flanked by adenosines, indicates that thousands of human genes are microRNA targets.. Cell.

[4] Kumar MS, Lu J, Mercer KL, Golub TR, Jacks T (2007). Impaired microRNA processing enhances cellular transformation and tumorigenesis.. Nat Genet.

[5] Hu Z, Chen J, Tian T, Zhou X, Gu H (2008). Genetic variants of miRNA sequences and non-small cell lung cancer survival.. J Clin Invest.

[6] Pineau P, Volinia S, McJunkin K, Marchio A, Battiston C, et al. miR-221 overexpression contributes to liver tumorigenesis.. Proc Natl Acad Sci U S A.

[7] Chen K, Song F, Calin GA, Wei Q, Hao X (2008). Polymorphisms in microRNA targets: a gold mine for molecular epidemiology.. Carcinogenesis.

[8] Hoffman AE, Zheng T, Yi C, Leaderer D, Weidhaas J (2009). microRNA miR-196a-2 and breast cancer: a genetic and epigenetic association study and functional analysis.. Cancer Res.

[9] Hu Z, Liang J, Wang Z, Tian T, Zhou X (2009). Common genetic variants in pre-microRNAs were associated with increased risk of breast cancer in Chinese women.. Hum Mutat.

[10] Catucci I, Yang R, Verderio P, Pizzamiglio S, Heesen L (2010). Evaluation of SNPs in miR-146a, miR196a2 and miR-499 as low-penetrance alleles in German and Italian familial breast cancer cases.. Hum Mutat.

[11] Tian T, Shu Y, Chen J, Hu Z, Xu L (2009). A functional genetic variant in microRNA-196a2 is associated with increased susceptibility of lung cancer in Chinese.. Cancer Epidemiol Biomarkers Prev.

[12] Kim MJ, Yoo SS, Choi YY, Park JY (2010). A functional polymorphism in the pre-microRNA-196a2 and the risk of lung cancer in a Korean population.. Lung Cancer.

[13] Qi P, Dou TH, Geng L, Zhou FG, Gu X (2010). Association of a variant in MIR 196A2 with susceptibility to hepatocellular carcinoma in male Chinese patients with chronic hepatitis B virus infection.. Hum Immunol.

[14] Li XD, Li ZG, Song XX, Liu CF (2010). A variant in microRNA-196a2 is associated with susceptibility to hepatocellular carcinoma in Chinese patients with cirrhosis.. Pathology.

[15] Peng S, Kuang Z, Sheng C, Zhang Y, Xu H (2010). Association of microRNA-196a-2 gene polymorphism with gastric cancer risk in a Chinese population.. Dig Dis Sci.

[16] Okubo M, Tahara T, Shibata T, Yamashita H, Nakamura M (2010). Association Between Common Genetic Variants in Pre-microRNAs and Gastric Cancer Risk in Japanese Population.. Helicobacter.

[17] Ye Y, Wang KK, Gu J, Yang H, Lin J (2008). Genetic variations in microRNA-related genes are novel susceptibility loci for esophageal cancer risk.. Cancer Prev Res (Phila).

[18] Wang K, Guo H, Hu H, Xiong G, Guan X (2010). A functional variation in pre-microRNA-196a is associated with susceptibility of esophageal squamous cell carcinoma risk in Chinese Han.. Biomarkers.

[19] Dou T, Wu Q, Chen X, Ribas J, Ni X (2010). A polymorphism of microRNA196a genome region was associated with decreased risk of glioma in Chinese population.. J Cancer Res Clin Oncol.

[20] Srivastava K, Srivastava A, Mittal B (2010). Common genetic variants in pre-microRNAs and risk of gallbladder cancer in North Indian population.. J Hum Genet.

[21] George GP, Gangwar R, Mandal RK, Sankhwar SN, Mittal RD (2011). Genetic variation in microRNA genes and prostate cancer risk in North Indian population.. Mol Biol Rep.

[22] Liu Z, Li G, Wei S, Niu J, El-Naggar AK (2010). Genetic variants in selected pre-microRNA genes and the risk of squamous cell carcinoma of the head and neck.. Cancer.

[23] Gao LB, Bai P, Pan XM, Jia J, Li LJ (2011). The association between two polymorphisms in pre-miRNAs and breast cancer risk: a meta-analysis.. Breast Cancer Res Treat.

[24] Christensen BC, Avissar-Whiting M, Ouellet LG, Butler RA, Nelson HH (2010). Mature microRNA sequence polymorphism in MIR196A2 is associated with risk and prognosis of head and neck cancer.. Clin Cancer Res.

[25] Lau J, Ioannidis JP, Schmid CH (1997). Quantitative synthesis in systematic reviews.. Ann Intern Med.

[26] DerSimonian R, Laird N (1986). Meta-analysis in clinical trials.. Control Clin Trials.

[27] Mantel N, Haenszel W (1959). Statistical aspects of the analysis of data from retrospective studies of disease.. J Natl Cancer Inst.

[28] Whitehead A, Whitehead J (1991). A general parametric approach to the meta-analysis of randomized clinical trials.. Stat Med.

[29] Egger M, Davey Smith G, Schneider M, Minder C (1997). Bias in meta-analysis detected by a simple, graphical test.. Bmj.

[30] Kim VN, Nam JW (2006). Genomics of microRNA.. Trends Genet.

[31] Bartel DP (2004). MicroRNAs: genomics, biogenesis, mechanism, and function.. Cell.

[32] Harfe BD (2005). MicroRNAs in vertebrate development.. Curr Opin Genet Dev.

[33] Duan R, Pak C, Jin P (2007). Single nucleotide polymorphism associated with mature miR-125a alters the processing of pri-miRNA.. Hum Mol Genet.

[34] Ryan BM, Robles AI, Harris CC. Genetic variation in microRNA networks: the implications for cancer research.. Nat Rev Cancer.

[35] Landgraf P, Rusu M, Sheridan R, Sewer A, Iovino N (2007). A mammalian microRNA expression atlas based on small RNA library sequencing.. Cell.

[36] Hirschhorn JN, Lohmueller K, Byrne E, Hirschhorn K (2002). A comprehensive review of genetic association studies.. Genet Med.

